# The association of growth differentiation factor 5 rs143383 gene polymorphism with osteoarthritis: a systematic review and meta-analysis

**DOI:** 10.1186/s13018-023-04245-y

**Published:** 2023-10-10

**Authors:** Yue-peng Wang, Wen-jia Di, Su Yang, Shi-lei Qin, Yun-feng Xu, Peng-fei Han, Ke-dong Hou

**Affiliations:** 1 Department of Orthopaedics, Beijing Friendship Hospital Pinggu Campus, Capital Medical University, Beijing, 101200 China; 2 Department of Graduate School, Baotou Medical College, Inner Mongolia University of Science and Technology, Baotou, 014040 China; 3 Department of Orthopaedics, Heping Hospital Affiliated to Changzhi Medical College, Changzhi, 046000 China; 4 Department of Orthopaedics, Changzhi Yunfeng Hospital, Changzhi, 046000 China

**Keywords:** Osteoarthritis, GDF5, rs143383, Single nucleotide polymorphism, Meta-analysis

## Abstract

**Background:**

Osteoarthritis (OA) is caused by a complex set of pathophysiological factors. The genetic factors involved in the occurrence and progress of the disease have been widely discussed by scholars. It was found that growth differentiation factor 5 (GDF5) gene polymorphisms may be linked to OA susceptibility, which has been controversial and needs to be further confirmed by an updated meta-analysis.

**Objectives:**

We examined the association between GDF5 rs143383 single nucleotide polymorphism (SNP) and OA susceptibility.

**Methods:**

All relevant articles that met the criteria are retrieved and included, and the search deadline is June 2022. The allele frequencies and different genotype frequencies of GDF5 rs143383 loci in each study were extracted and statistically analyzed by R4.1.3 software, and the different genetic models were analyzed based on their odds ratio (OR) and 95% confidence interval (CI).

**Results:**

The meta-analysis explained that GDF5 rs143383 SNP was crucial correlated with OA in all patients with OA of knee, hip and hand. The codominant gene model in the whole crowd (OR = 1.17, 95% CI 1.07–1.27, *P* < 0.01) enlightened that OA was vitally associated with GDF5 gene polymorphism. At the same time, we did a subgroup analysis based on ethnicity. The codominant gene model (OR = 1.31, 95% CI 1.12–1.53, *P* < 0.01) in Asian population, the codominant homozygote model (OR = 1.28, 95% CI 1.14–1.43), codominant heterozygote gene model (OR = 1.12, 95% CI 1.01–1.23, *P* = 0.02), and dominant gene model (OR = 1.19, 95% CI 1.09–1.31, *P* < 0.01) in Caucasian are analyzed by subgroup analysis. It means that there is a momentous relationship between the GDF5rs143383 gene polymorphism and OA, especially among Caucasians. In addition, we also discussed different types of OA separately and discover that the GDF5rs143383 gene polymorphism was relevant for knee osteoarthritis (KOA) and hand osteoarthritis, and it was more significant in the Caucasian population. But due to the high heterogeneity in hip osteoarthritis, it could not be accurately concluded. Furthermore, we also analyzed the osteoarthritis of different genders and found that the GDF5 rs143383 SNP was associated with both men and women and was still significant in the Caucasian population.

**Conclusion:**

We found a close association between osteoarthritis and GDF5rs143383SNP in this study. From the analysis of each group, we got the same conclusion in KOA and hand OA, but which need further verification in hip OA. Considering gender, we found a close relationship between GDF5 rs143383 SNP and OA of the knee, hip and hand, both for men and women. This conclusion is more obvious in Caucasian people.

## Introduction

Osteoarthritis (OA), also considered as chronic arthritis, refers to a degenerative process in the articular cartilage of the joints, subchondral bone reactive hyperplasia, inflammation and osteophyte formation based on joint degeneration or aging, and characterized by joint swelling, pain or dysfunction [1]. OA is a chronic bone and joint disease caused by cartilage degeneration and bone hyperplasia of the joint, also known as proliferative arthritis, degenerative arthritis and osteoarthritis. This disease mostly occurs in the elderly, but also in young people [2]. OA is a major disease that causes joint pain and limited activity in the elderly, and Middle-aged and elderly people suffer from serious health problems as a result of it. Several studies have found that age, joint trauma, obesity and genetic susceptibility are risk factors for OA [3, 4]. Growth differentiation factor 5 (GDF5) is also known as CDMP-1 and BMP-14. It is the growth differentiation factor that regulates tissue growth, and it is bone morphogenetic protein and a member of the transforming growth factor β family, which plays a crucial role in the progression, protection and rehabilitate of bone and cartilage [5]. It has been reported that GDF5 gene mutations can directly lead to some bone-related diseases. The content of GDF5 increases in gradient in the area where cartilage precursor cells gather, the cartilage core where long bones develop, and the joint formation areas, thereby exerting its special biological functions, such as regulating limb bud cell development and maintaining cell dynamic balance [6]. Furthermore, GDF5 also regulates the proliferation and differentiation of limb bud cells in the embryonic stage. In view of GDF5’s vital function, OA is considered to be related to it [7–9]. Some previous studies considered the correlation between GDF5 and OA, but some studies still believe that there is no significant correlation.

A number of shortcomings have been found in previous meta-analyses, including incorrect data extraction and insufficient analysis of population subgroups. Additionally, some literature has been updated. The main purpose of this article is to make a comprehensive analysis of the association between GDF5 rs143383 SNP and OA in different types of osteoarthritis, such as knee osteoarthritis, hip osteoarthritis and hand osteoarthritis. At the same time, from the analysis of gender and different nationalities, we can get the most accurate conclusion at present, which can be used to guide clinical work and carry out related drug research.

## Materials and methods

### Search strategy

This study conformed to PRISMA guidelines. The databases searched included PubMed, Cochrane Library, the Web of Science, EMBASE, China National Knowledge Infrastructure (CNKI), Wanfang Data Knowledge Service Platform and other databases to investigate the correlation between GDF5 rs143383 SNP and OA. In terms of search strategy, we used (“growth and differentiation factor 5” or “GDF5” or “rs143383”) and (“SNP” or “polymorphism”) and (“OA” or “osteoarthritis”). The language is limited to English and Chinese, and the time limit for retrieval is from the institution of the database to June 2022.

### Selection criteria

Selection criteria for the review included the following:

1. Case–control study.
2. The case group meets the diagnostic criteria of OA (the diagnosis of OA was based on the American College of Rheumatology criteria), and the control group was healthy.
3. The full text of the original literature is available, involving GDF5 rs143383, and there are specific data on sample size, genotype and gene frequency in the case group and the control group.

The following criteria were used to exclude studies:

1. Other observational study designs include pedigree correlation studies, case reports, clinical trials, reviews and comments.
2. The case group was not in accordance with the OA diagnosis, and the control group was not healthy.
3. GDF5 rs143383 are not the SNP of interest, or OA is not the phenotype of interest.

### Data extraction

The literature has been read by both authors in its entirety and according to the selection criteria. They searched independently the above-mentioned databases and extracted the information included in the literature. We did not include literature that could not be retrieved from the database or that was undergoing review and for which we were unable to retrieve it. The authors of this article were blinded to the authors and institutions of the studies undergoing review. Finally, data were thoroughly analyzed and extracted from all relevant studies, including a comprehensive search and a comprehensive information extraction process. When collecting data to initially screen the literature, a large number of articles are included, and there may be inconsistencies in the screening results between the two authors. In this case, our two authors will screen the literature again in strict accordance with the inclusion and exclusion criteria. Or read the full text of the article carefully with the other authors, and accurately screen out the literature that meets the inclusion and exclusion criteria listed in this article, in order to ensure the accuracy of the article.

The following information was extracted from studies included in reviewing:

1. First author’s last name;
2. The year of publication;
3. A description of the study’s country of source;
4. Ethnicity;
5. Sample size, genotypes and alleles of the OA group and the control group.

### Quality assessment

We applied the modified Newcastle–Ottawa scale (NOS) to review the literature on the relevance between GDF5 rs143383 SNP and OA included in the study and assess its quality. The modified NOS has a total of 9 stars and includes three aspects: selection, comparability and outcome. When ≥ 5 stars, it can be regarded as good quality, and it may be necessary to adjust the relative threshold depending on the technology used.

### Credibility analysis

R4.1.3 software is used to analyze the extracted data by Meta. Two-classified variables are expressed by the odds ratio (OR) and 95% confidence interval (CI). We calculate, respectively, the OR and 95% CI of the GDF5 rs143383 allele model (T vs. C), codominant homozygote model (TT vs. CC), codominant heterozygote model (TC vs. CC), dominant model (TT + TC vs. CC) and recessive model (TT vs. TC + CC), and statistics were considered significant when *P* values were less than 0.05. It is necessary to determine whether gene frequency in the literature is consistent with Hardy–Weinberg equilibrium (HWE). If *P* < 0. 05, the gene frequency distribution of the control group does not accord with HWE, and if *P* > 0. 05, the gene frequency distribution of the control group accords with HWE. According to ethnicity, the included population was divided into Asian and Caucasian subgroups for analysis. Heterogeneity is evaluated by *I*^2^: *I*^2^ < 50%, the heterogeneity is small, and fixed effect model is used; *I*^2^ ≥ 50%, heterogeneity is large, and random effect model is used.

## Results

### Literature retrieval results

**Fig. 1:**
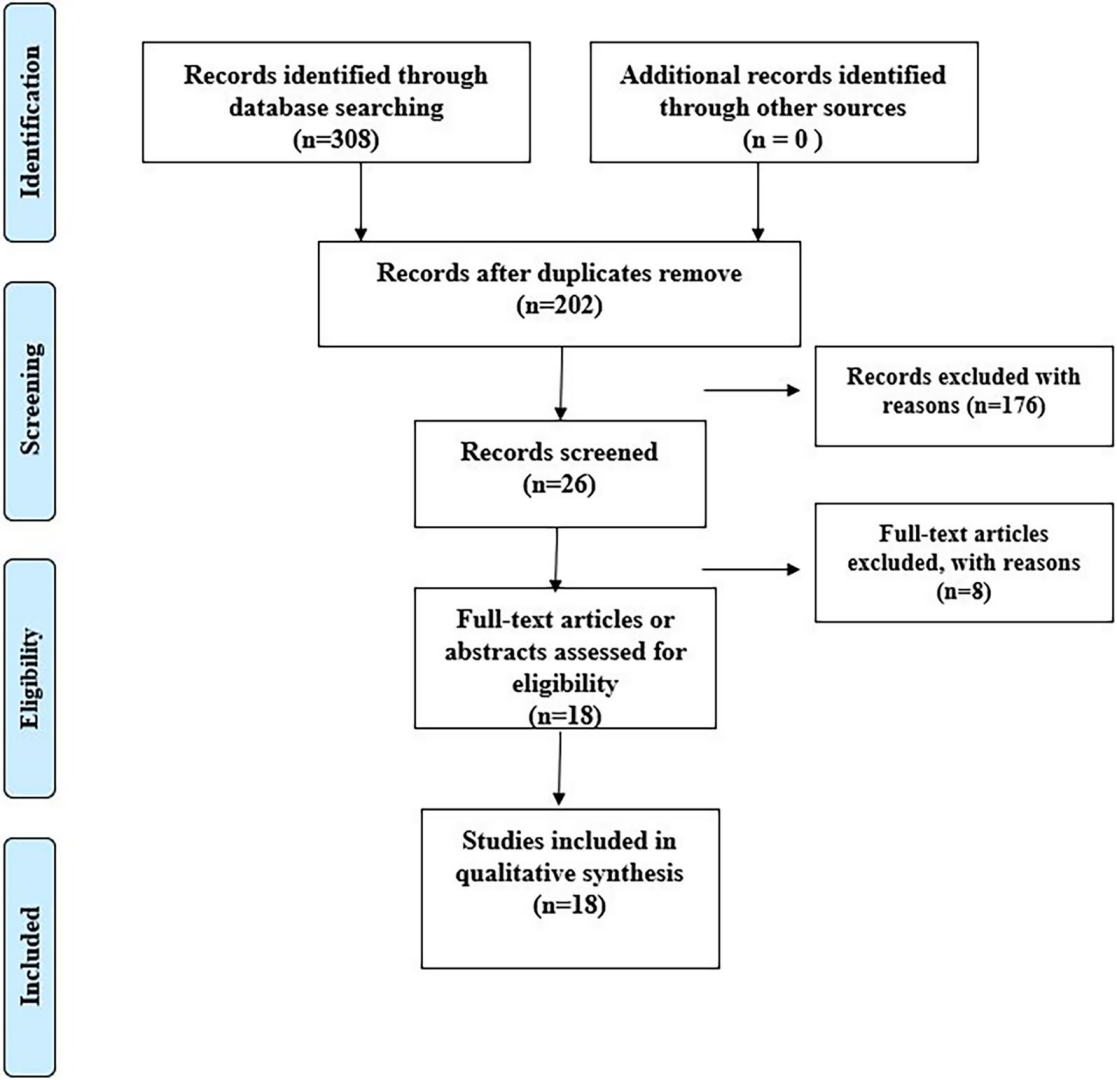
Flow diagram of the literature search

According to the above retrieval strategy, a total of 308 associated articles were searched. Based on the abstracts and titles of the papers, repetition and articles unrelated to the study’s objectives were excluded. We screened 26 articles related to the topic, and inclusion and exclusion criteria were strictly followed throughout the entire text. Finally, 17 foreign articles and 1 Chinese article were included, and the 18 articles included 12,060 patients with OA (case group) and 18,401 controls (control group). As shown in Fig. 1, the literature screening procedure and outcome as well as the basic characteristics included in literature research are listed in Table 1.

**Table 1:** The basic characteristics of the study included

Study	Year	Country	Ethnicity	OA	OA/Control	Male/Female	OA			Control			NOS score
							TT	TC	CC	TT	TC	CC	
Miyamoto [10]	2007	Japan	Asian	Knee\Hip	1716/1844	NA/NA	1145	509	62	1015	701	128	6
Southam [11]	2007	Spain and UK	Caucasian	Knee\Hip\Hand	2487/2018	1845/2658	974	1194	319	763	935	320	7
Tsezou [12]	2008	Greece	Caucasian	Knee	251/267	144/374	95	126	30	99	125	44	7
Yao [13]	2008	China	Asian	Knee	313/485	275/523	197	97	19	244	193	48	8
Chapman [14]	2008	Netherland	Caucasian	Knee\Hip\Hand	363/724	NA/NA	121	189	53	289	331	104	7
Vaes [15]	2009	Netherland	Caucasian	Knee\Hip\Hand	1824/7034	3732/5126	754	820	250	2582	3353	1099	8
Valdes [16]	2009	UK	Caucasian	Knee\Hip	1858/1155	NA/NA	840	777	241	419	573	163	7
Cao [17]	2010	Korea	Asian	Knee	276/298	213/361	150	115	11	159	113	26	7
Takahash [18]	2010	Japan	Asian	Knee	933/1225	477/1681	566	313	54	684	461	80	7
Tawonsawatruk [19]	2011	Thailand	Asian	Knee	90/103	21/172	38	41	11	33	47	23	7
Shin [20]	2012	Korea	Asian	Knee	725/1737	1035/1409	382	305	38	942	689	106	8
Elazeem [21]	2017	Egypt	Caucasian	Knee	50/50	18/82	20	16	14	12	25	13	8
Mishra [22]	2017	India	Asian	Knee	500/500	429/571	199	226	75	131	272	97	6
Ozcan [23]	2017	Turkey	Caucasian	Knee	94/279	NA/NA	37	43	14	74	153	52	7
García-Alvarado [24]	2018	Mexico	Caucasian	Knee	145/145	144/146	87	51	7	66	65	14	7
Mohasseb [25]	2019	Egypt	Caucasian	Knee	47/40	18/69	14	23	10	16	13	11	8
Zhang [26]	2019	China	Asian	Knee	288/397	314/371	124	105	59	206	159	32	6
Moghimi [27]	2021	Iran	Asian	Knee\Hand	100/100	50/1150	52	34	14	12	46	42	7

### Quality evaluation result of included literature

The quality of the included literature was assessed by the modified NOS. Research quality score of 17 articles was all above 5 stars (Table 1), and the overall quality was higher. All the inclusion researches were case–control studies, and there was no exclusion or inclusion criteria that they did not meet.

### Meta-analysis results

Most of meta-analysis results are *P* < 0.05. However, the high heterogeneity may lead to uncertainty in the results. We only reliably analyze the data with *I*^2^ < 50% to draw accurate conclusions.

### OA of knee, hip and hand

**Fig. 2:**
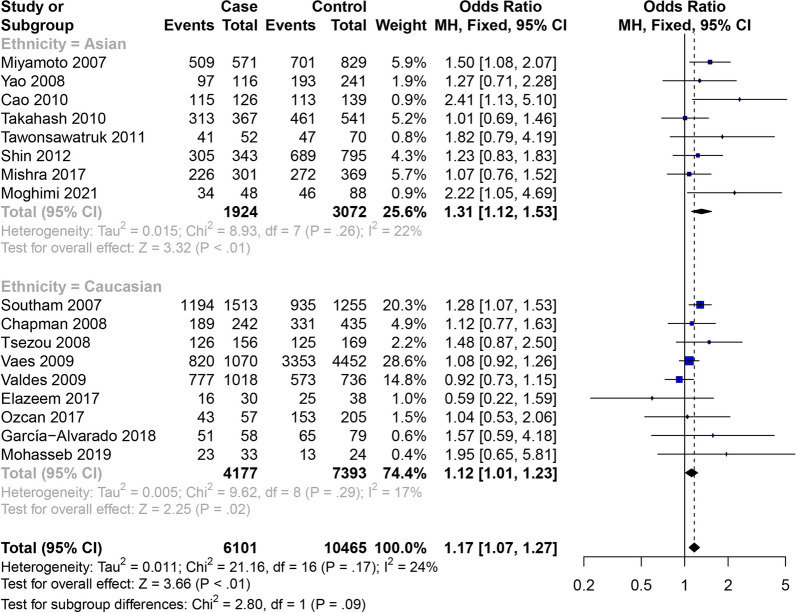
Forest plot of the correlation between GDF5 gene polymorphism and OA of knee, hip and hand risk. Codominant model (TC vs. CC)

All eighteen studies [10–27] presented the OA of knee, hip and hand data. In the studies of the correlation between the GDF5 rs143383 SNP and OA of knee, hip and hand, the meta-analysis showed that GDF5 rs143383 SNP codominant heterozygote model (OR = 1.17, 95% CI 1.07–1.27) is associated with the susceptibility to OA of knee, hip and hand. Conclusions of subgroup analysis proved that sensibility to OA of knee, hip and hand is associated with codominant heterozygote model (OR = 1.31, 95% CI 1.12–1.53) in the Asian population, while in Caucasian population codominant homozygote model (OR = 1.28, 95% CI 1.14–1.43), codominant heterozygote model (OR = 1.12, 95% CI 1.01–1.23) and dominant model (OR = 1.19, 95% CI 1.09–1.31) are susceptibility factors to OA of knee, hip and hand (Fig. 2). The specific results are shown in Table 4.

### Knee OA

**Fig. 3:**
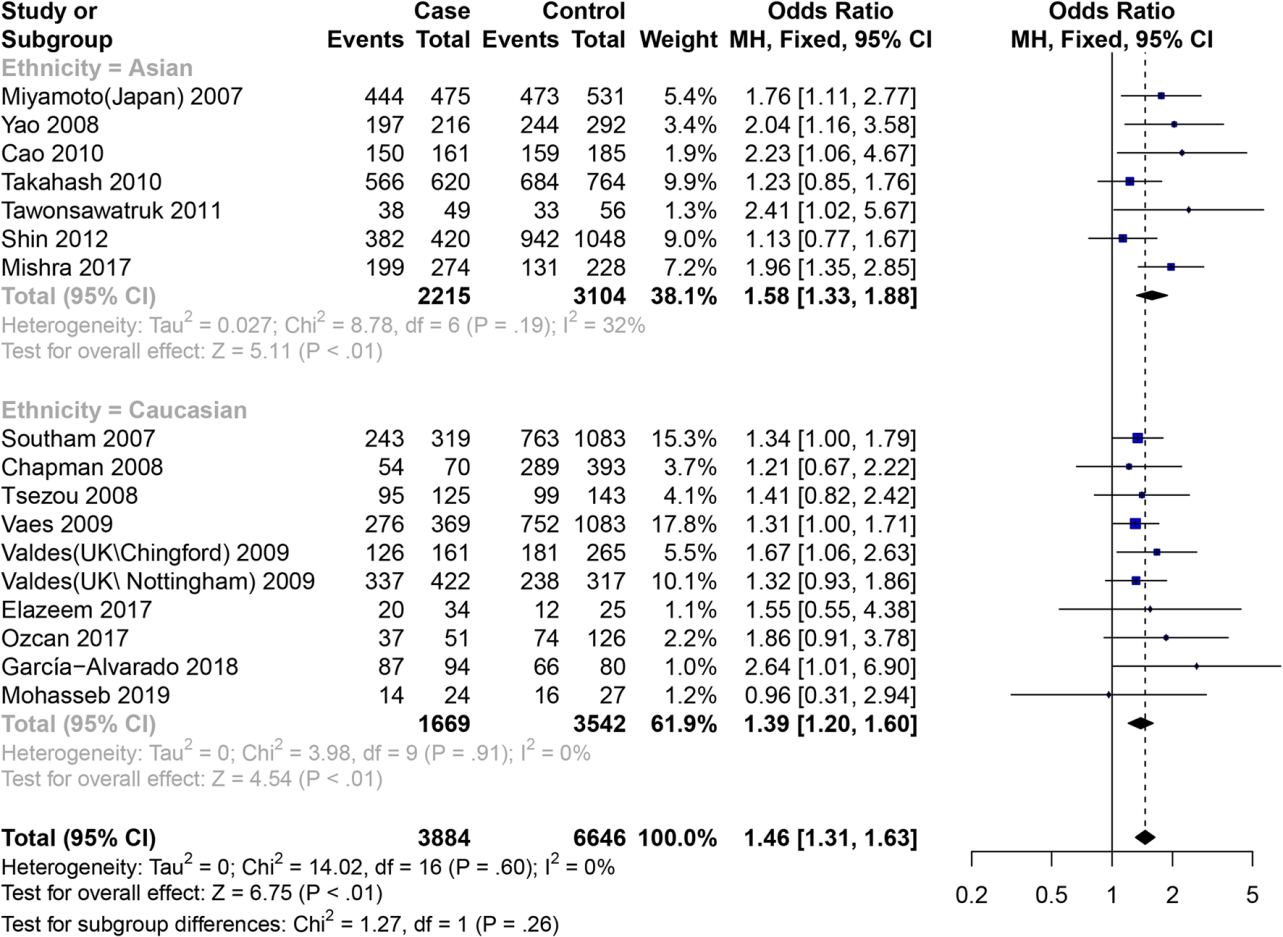
Forest plot of the correlation between GDF5 gene polymorphism and knee OA risk. Codominant model (TT vs. CC)

**Fig. 4:**
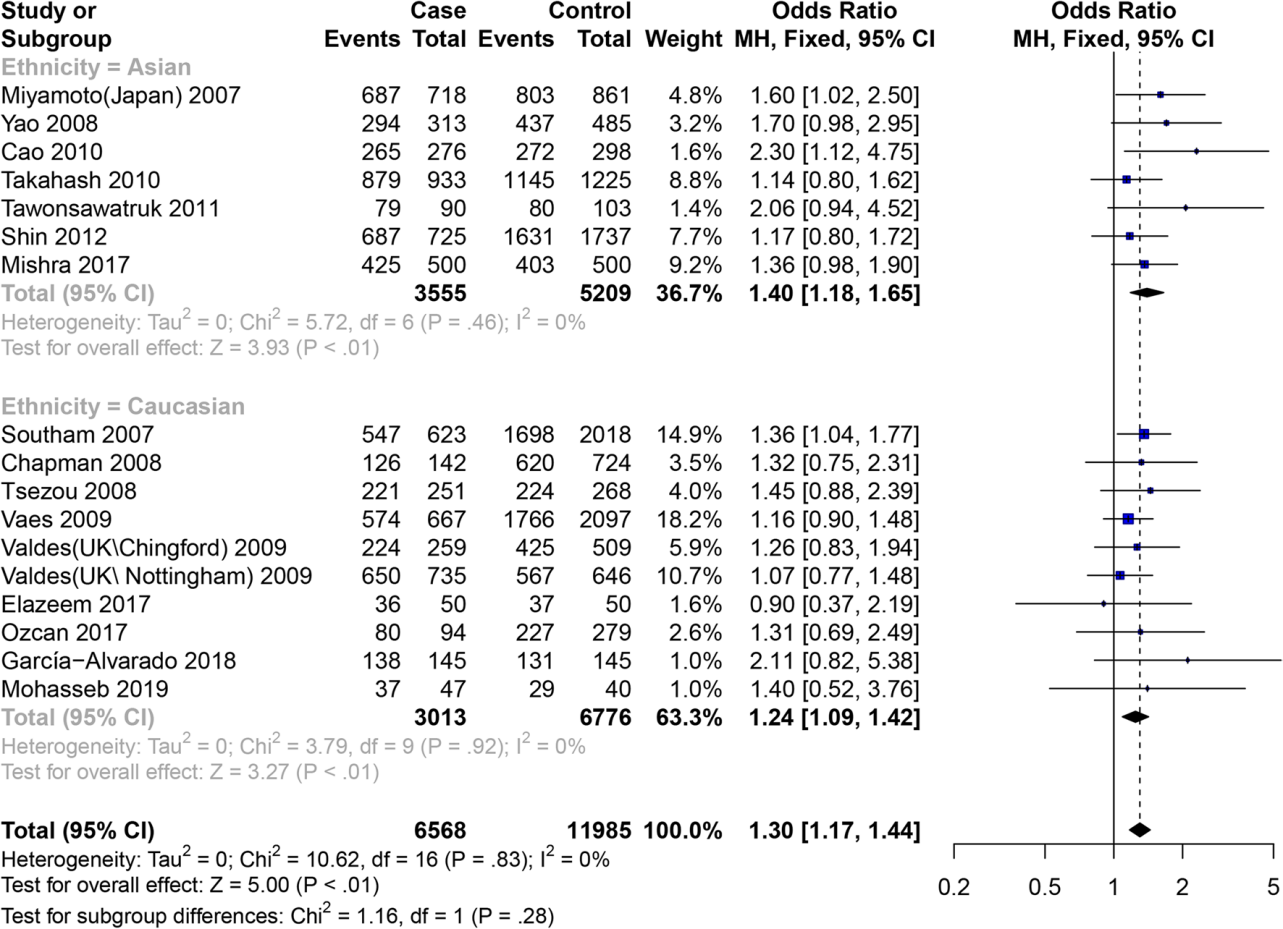
Forest plot of the correlation between GDF5 gene polymorphism and knee OA risk. Dominant model (TT + TC vs. CC)

Among the 17 articles [10–26], 18 items of data studied the connection between GDF5 rs143383 SNP and KOA (Table 2). The total meta-analysis explained that the susceptiveness to KOA is associated with GDF5 rs143383 SNP allele model, codominant homozygote model, codominant heterozygote model and dominant model. Analyzing subgroups revealed the following that codominant homozygote model (OR = 1.58, 95% CI 1.33–1.88), codominant heterozygote model (OR = 1.23, 95% CI 1.03–1.46) and dominant model (OR = 1.40, 95% CI 1.18–1.65) are associated with the susceptibility to KOA in the Asian population. We also found that among the Caucasian population allele model (OR = 1.19, 95% CI 1.12–1.27), codominant homozygote model (OR = 1.39, 95% CI 1.20–1.60) and dominant model (OR = 1.24, 95% CI 1.09–1.42) are associated with the susceptibility to KOA, while there is no statistical significance in the codominant heterozygote genes (OR = 1.14, 95% CI 0.99–1.30) (Figs. 3 and 4). The specific results are shown in Table 4.

**Table 2:** Characteristics of the included studies for osteoarthritis of the knee, hip and hand

Study	Year	Country	Ethnicity	OA/Control	Male/Female	OA			Control		
						TT	TC	CC	TT	TC	CC
*Knee*											
Miyamoto	2007	Japan	Asian	718/861	NA/NA	444	243	31	473	330	58
Southam	2007	Spain and UK	Caucasian	623/2018	1204/1437	243	304	76	763	935	320
Chapman	2008	Netherland	Caucasian	142/724	NA/NA	54	72	16	289	331	104
Tsezou	2008	Greece	Caucasian	251/267	144/374	95	126	30	99	125	44
Yao	2008	China	Asian	313/485	275/523	197	97	19	244	193	48
Vaes	2009	Netherland	Caucasian	667/2097	1096/1668	276	298	93	752	1014	331
Valdes	2009	UK (Chingford)	Caucasian	259/509	NA/NA	126	98	35	181	244	84
Valdes	2009	UK (Nottingham)	Caucasian	735/646	NA/NA	337	313	85	238	329	79
Cao	2010	Knee	Korea	Asian	276/298	150	115	11	431	165	159
Takahash	2010	Japan	Asian	933/1225	477/1681	566	313	54	684	461	80
Tawonsawatruk	2011	Thailand	Asian	90/103	21/172	38	41	11	33	47	23
Shin	2012	Korea	Asian	725/1737	1035/1409	382	305	38	942	689	106
Elazeem	2017	Egypt	Caucasian	50/50	18/82	20	16	14	12	25	13
Mishra	2017	India	Asian	500/500	429/571	199	226	75	131	272	97
Ozcan	2017	Turkey	Caucasian	94/279	NA/NA	37	43	14	74	153	52
García-Alvarado	2018	Mexico	Caucasian	145/145	144/146	87	51	7	66	65	14
Mohasseb	2019	Egypt	Caucasian	47/40	18/69	14	23	10	16	13	11
Zhang	2019	China	Asian	288/397	314/371	124	105	59	206	159	32
*Hip*											
Miyamoto	2007	Japan	Asian	998/983	NA/NA	701	266	31	542	371	70
Southam	2007	Spain and UK	Caucasian	1525/2018	1560/1983	599	728	198	763	935	320
Chapman	2008	Netherland	Caucasian	106/724	NA/NA	43	50	13	289	331	104
Vaes	2009	Netherland	Caucasian	287/2757	1292/1752	111	131	45	1040	1298	419
Valdes	2009	UK (Chingford)	Caucasian	77/509	NA/NA	32	27	18	181	244	84
Valdes	2009	UK (Nottingham)	Caucasian	787/646	NA/NA	345	339	103	238	329	79
*Hand*											
Southam	2007	Spain and UK	Caucasian	240*/2018	1010/1246	98	105	37	763	935	320
Chapman	2008	Netherland	Caucasian	200/724	NA/NA	64	111	25	289	331	104
Vaes	2009	Netherland	Caucasian	870/2180	1344/1706	367	391	112	790	1041	349

^*^Two hand osteoarthritis cases had unrecorded sex status

### Hip OA

Five articles [10, 11, 14–16] (6 items of data) studied the relativity between the GDF5 rs143383 SNP and hip OA (Table 2), and the overall heterogeneity is high and it is impossible to draw an accurate conclusion. Through the heterogeneity analysis of the subgroup, it was found that the heterogeneity came from the Asian population group, so only the Caucasian population was analyzed. The outcome of subgroup analysis explicated that in Caucasian population allele model (OR = 1.08, 95% CI 1.01–1.16) and recessive model (OR = 1.12, 95% CI 1.02–1.24) are related to the susceptibility to hip OA, while there is no statistical significance in the codominant homozygote model (OR = 1.15, 95% CI 0.99–1.34) and dominant model (OR = 1.09, 95% CI 0.95–1.26). The specific results are shown in Table 4.

### Hand OA

**Fig. 5:**
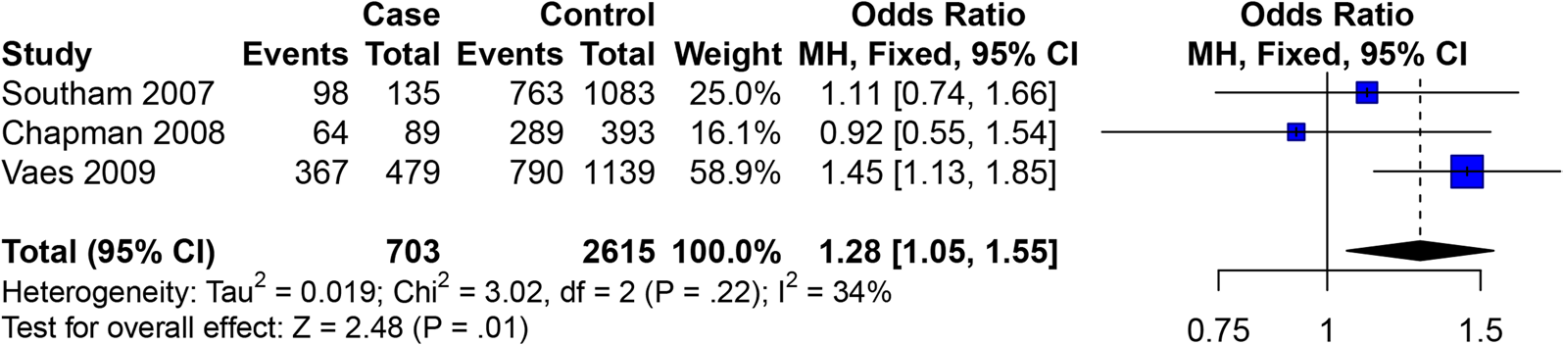
Forest plot of the correlation between GDF5 gene polymorphism and hand OA risk. Codominant model (TT vs. CC)

In the studies [11, 14, 15] of the relevance between the GDF5 rs143383 SNP and hand OA (Table 2), the meta-analysis showed that GDF5 rs143383 SNP codominant homozygote model (OR = 1.28, 95% CI 1.05–1.55) and dominant model (OR = 1.09, 95% CI 0.95–1.26) are associated with the susceptibility to hand OA, while there is no statistical significance in the codominant heterozygote model (OR = 1.15, 95% CI 0.95–1.40) **(**Fig. 5**)**. The specific results are shown in Table 4.

### Male OA of knee, hip and hand

**Fig. 6:**
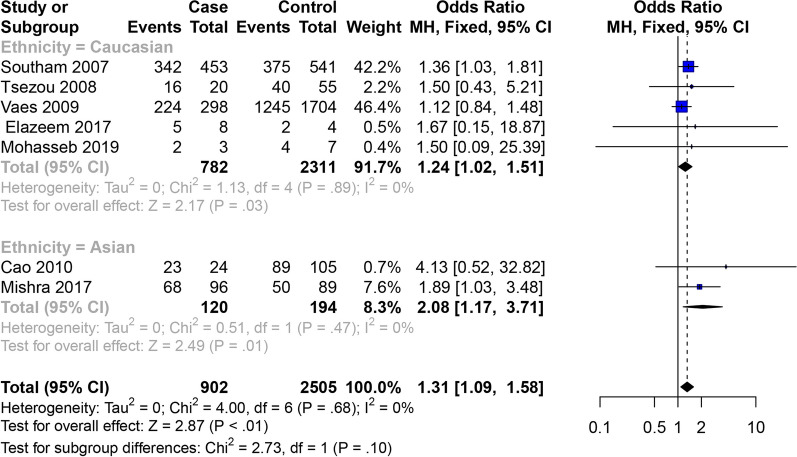
Forest plot of the correlation between GDF5 gene polymorphism and male OA of knee, hip and hand risk. Codominant model (TT vs. CC)

**Fig. 7:**
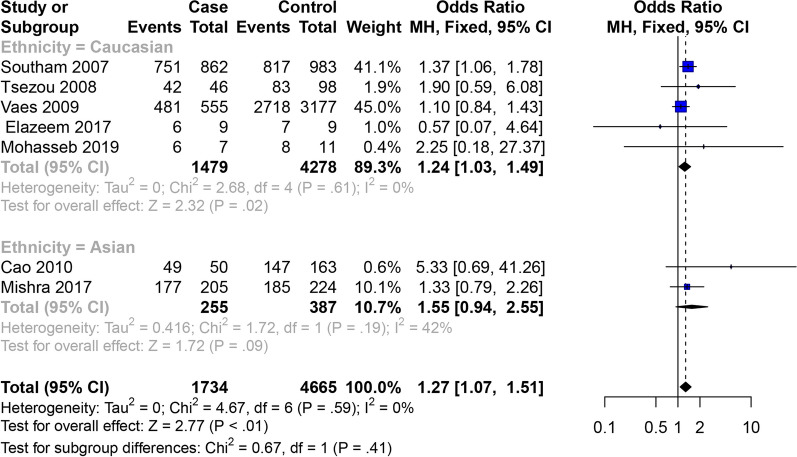
Forest plot of the correlation between GDF5 gene polymorphism and male OA of knee, hip and hand risk. Dominant model (TT + TC vs. CC)

Eight articles studied the relativity between the GDF5 rs143383 SNP and male OA (Table 3). The overall meta-analysis showed that GDF5 rs143383 SNP allele model, codominant homozygote model, codominant heterozygote model and dominant model are associated with the susceptibility to male OA, while there is no statistical significance in the recessive model. Analyses of subgroups revealed the following: codominant homozygote model (OR = 2.08, 95% CI 1.17–3.71) is related to the susceptibility to OA in the Asian males, while it is no statistical significance in allele model (OR = 1.25, 95% CI 0.99–1.59) and dominant model (OR = 1.55, 95% CI 0.94–2.55). The codominant homozygote model (OR = 1.24, 95% CI 1.02–1.51), codominant heterozygote model (OR = 1.24, 95% CI 1.02–1.50) and dominant model (OR = 1.24, 95% CI 1.03–1.49) are related to the susceptibility to OA in the Caucasian males, while it is no statistical significance in allele model (OR = 1.09, 95% CI 0.99–1.19) and recessive model (OR = 1.05, 95% CI 0.93–1.20) (Figs. 6 and 7). The specific results are shown in Table 4.

**Table 3:** Characteristics of the included studies for osteoarthritis of sex

Study	Year	Country	Ethnicity	OA/Control	OA			Control		
					TT	TC	CC	TT	TC	CC
*Male*										
Southam	2007	Spain and UK	Caucasian	862/983	342	409	111	375	442	166
Tsezou	2008	Greece	Caucasian	46/98	16	26	4	40	43	15
Vaes	2009	Netherland	Caucasian	555/3177	224	257	74	1245	1473	459
Cao	2010	Korea	Asian	50/163	23	26	1	89	58	16
Elazeem	2017	Egypt	Caucasian	9/9	5	1	3	2	5	2
Mishra	2017	India	Asian	205/224	68	109	28	50	135	39
Mohasseb	2019	Egypt	Caucasian	7/11	2	4	1	4	4	3
Zhang	2019	China	Asian	129/185	56	47	26	97	74	14
*Female*										
Southam	2007	Spain and UK	Caucasian	1623/1035	342	409	111	375	442	166
Tsezou	2008	Greece	Caucasian	205/169	16	26	4	40	43	15
Vaes	2009	Netherland	Caucasian	1269/3857	224	257	74	1245	1473	459
Cao	2010	Korea	Asian	226/135	23	26	1	89	58	16
Elazeem	2017	Egypt	Caucasian	41/41	5	1	3	2	5	2
Mishra	2017	India	Asian	295/276	68	109	28	50	135	39
Mohasseb	2019	Egypt	Caucasian	40/29	2	4	1	4	4	3
Zhang	2019	China	Asian	159/212	68	58	33	109	85	18

**Table 4:** The analysis results of genetic models on the association of GDF5 rs143383 polymorphism with OA

Allele gene and genotype	OR	95% CI	*P* (%)	Model	*I*^2^ (%)
OA of knee, hip and hand					
Overall					
T vs. C	1.28	1.15–1.42	*P* < 0.01	R	83
TT vs. CC	1.61	1.33–1.94	*P* < 0.01	R	73
**TC vs. CC**	**1.17**	**1.07–1.27**	***P* < 0.01**	**F**	**24**
TT + TC vs. CC	1.37	1.20–1.57	*P* < 0.01	R	52
TT vs. TC + CC	1.34	1.16–1.56	*P* < 0.01	R	84
Asian					
T vs. C	1.46	1.19–1.78	*P* < 0.01	R	88
TT vs. CC	2.13	1.46–3.10	*P* < 0.01	R	79
**TC vs. CC**	**1.31**	**1.12–1.53**	***P* < 0.01**	**F**	**22**
TT + TC vs. CC	1.69	1.30–2.21	*P* < 0.01	R	62
TT vs. TC + CC	1.55	1.20–2.00	*P* < 0.01	R	88
Caucasian					
T vs. C	1.14	1.04–1.26	*P* = 0.01	R	65
**TT vs. CC**	**1.28**	**1.14–1.43**	***P* < 0.01**	**F**	**10**
**TC vs. CC**	**1.12**	**1.01–1.23**	***P* = 0.02**	**F**	**17**
**TT + TC vs. CC**	**1.19**	**1.09–1.31**	***P* < 0.01**	**F**	**0**
TT vs. TC + CC	1.19	1.01–1.41	*P* = 0.04	R	75
Knee OA					
Overall					
**T vs. C**	**1.20**	**1.15–1.26**	***P* < 0.01**	**F**	**44**
**TT vs. CC**	**1.46**	**1.31–1.63**	***P* < 0.01**	**F**	**0**
**TC vs. CC**	**1.17**	**1.05–1.30**	***P* < 0.01**	**F**	**1**
**TT + TC vs. CC**	**1.30**	**1.17–1.44**	***P* < 0.01**	**F**	**0**
TT vs. TC + CC	1.30	1.15–1.46	*P* < 0.01	R	65
Asian					
T vs. C	1.22	1.13–1.30	*P* < 0.01	R	68
**TT vs. CC**	**1.58**	**1.33–1.88**	***P* < 0.01**	**F**	**32**
**TC vs. CC**	**1.23**	**1.03–1.46**	***P* = 0.02**	**F**	**0**
**TT + TC vs. CC**	**1.40**	**1.18–1.65**	***P* < 0.01**	**F**	**0**
TT vs. TC + CC	1.31	1.08–1.59	*P* < 0.01	R	76
Caucasian					
**T vs. C**	**1.19**	**1.12–1.27**	***P* < 0.01**	**F**	**8**
**TT vs. CC**	**1.39**	**1.20–1.60**	***P* < 0.01**	**F**	**0**
TC vs. CC	1.14	0.99–1.30	*P* = 0.07	F	2
**TT + TC vs. CC**	**1.24**	**1.09–1.42**	***P* < 0.01**	**F**	**0**
TT vs. TC + CC	1.29	1.10–1.52	*P* < 0.01	R	57
Hip OA					
Overall					
T vs. C	1.17	0.97–1.41	*P* = 0.10	R	85
TT vs. CC	1.28	0.94–1.73	*P* = 0.11	R	72
TC vs. CC	1.02	0.78–1.34	*P* = 0.87	R	65
TT + TC vs. CC	1.15	0.86–1.53	*P* < 0.01	R	73
TT vs. TC + CC	1.26	1.00–1.59	*P* = 0.05	R	83
Caucasian					
**T vs. C**	**1.08**	**1.01–1.16**	***P* = 0.02**	**F**	**0**
TT vs. CC	1.15	0.99–1.34	*P* = 0.06	F	0
TC vs. CC	0.94	0.71–1.25	*P* = 0.68	R	64
TT + TC vs. CC	1.09	0.95–1.26	*P* = 0.21	F	43
**TT vs. TC + CC**	**1.12**	**1.02–1.24**	***P* = 0.02**	**F**	**1**
Hand OA					
T vs. C	1.07	0.89–1.28	*P* = 0.47	R	69
**TT vs. CC**	**1.28**	**1.05–1.55**	***P* = 0.01**	**F**	**34**
TC vs. CC	1.15	0.95–1.40	*P* = 0.14	F	0
**TT + TC vs. CC**	**1.21**	**1.01–1.45**	***P* = 0.04**	**F**	**0**
TT vs. TC + CC	1.04	0.75–1.43	*P* = 0.83	R	80
Male OA					
Overall					
**T vs. C**	**1.11**	**1.02–1.21**	***P* = 0.02**	**F**	**0**
**TT vs. CC**	**1.31**	**1.09–1.58**	***P* < 0.01**	**F**	**0**
**TC vs. CC**	**1.26**	**1.05–1.50**	***P* = 0.01**	**F**	**27**
**TT + TC vs. CC**	**1.27**	**1.07–1.51**	***P* < 0.01**	**F**	**0**
TT vs. TC + CC	1.08	0.96–1.22	*P* = 0.21	F	35
Asian					
T vs. C	1.25	0.99–1.59	*P* = 0.06	F	16
**TT vs. CC**	**2.08**	**1.17–3.71**	***P* = 0.01**	**F**	**0**
TC vs. CC	2.17	0.37–12.82	*P* = 0.39	R	67
TT + TC vs. CC	1.55	0.94–2.55	*P* = 0.09	F	42
TT vs. TC + CC	1.14	0.48–2.73	*P* = 0.76	R	81
Caucasian					
T vs. C	1.09	0.99–1.19	*P* = 0.08	F	0
**TT vs. CC**	**1.24**	**1.02–1.51**	***P* = 0.03**	**F**	**0**
**TC vs. CC**	**1.24**	**1.02–1.50**	***P* = 0.03**	**F**	**25**
**TT + TC vs. CC**	**1.24**	**1.03–1.49**	***P* = 0.02**	**F**	**0**
TT vs. TC + CC	1.05	0.93–1.20	*P* = 0.44	F	0
Female OA					
Overall					
**T vs. C**	**1.19**	**1.12–1.27**	***P* < 0.01**	**F**	**28**
**TT vs. CC**	**1.41**	**1.22–1.61**	***P* < 0.01**	**F**	**0**
TC vs. CC	1.14	0.99–1.30	*P* = 0.06	F	0
**TT + TC vs. CC**	**1.25**	**1.10–1.42**	***P* < 0.01**	**F**	**0**
TT vs. TC + CC	1.28	1.06–1.54	***P* < 0.01**	R	57
Asian					
**T vs. C**	**1.41**	**1.16–1.72**	***P* < 0.01**	**F**	**13**
**TT vs. CC**	**1.96**	**1.28–2.98**	***P* < 0.01**	**F**	**0**
TC vs. CC	1.14	0.76–1.72	*P* = 0.52	F	0
TT + TC vs. CC	1.46	0.99–2.14	*P* = 0.06	F	0
TT vs. TC + CC	1.54	0.96–2.46	*P* = 0.07	R	66
Caucasian					
**T vs. C**	**1.17**	**1.09–1.25**	***P* < 0.01**	**F**	**0**
**TT vs. CC**	**1.35**	**1.17–1.56**	***P* < 0.01**	**F**	**0**
TC vs. CC	1.14	0.99–1.31	*P* = 0.08	F	0
**TT + TC vs. CC**	**1.23**	**1.07–1.40**	***P* < 0.01**	**F**	**0**
**TT vs. TC + CC**	**1.22**	**1.11–1.35**	***P* < 0.01**	**F**	**49**

Statistical significance values are shown in bold (I2<50%)

R: random effect model; F: fixed effect model; P: corresponding to the Z test for the summary effect estimate (*P* < 0.05 considered statistically significant); *I*^2^: I^2^ = 0 no heterogeneity, *I*^2^ = 25% low heterogeneity, *I*^2^ = 50% moderate heterogeneity, and *I*^2^ = 75% high heterogeneity

### Female OA of knee, hip and hand

**Fig. 8:**
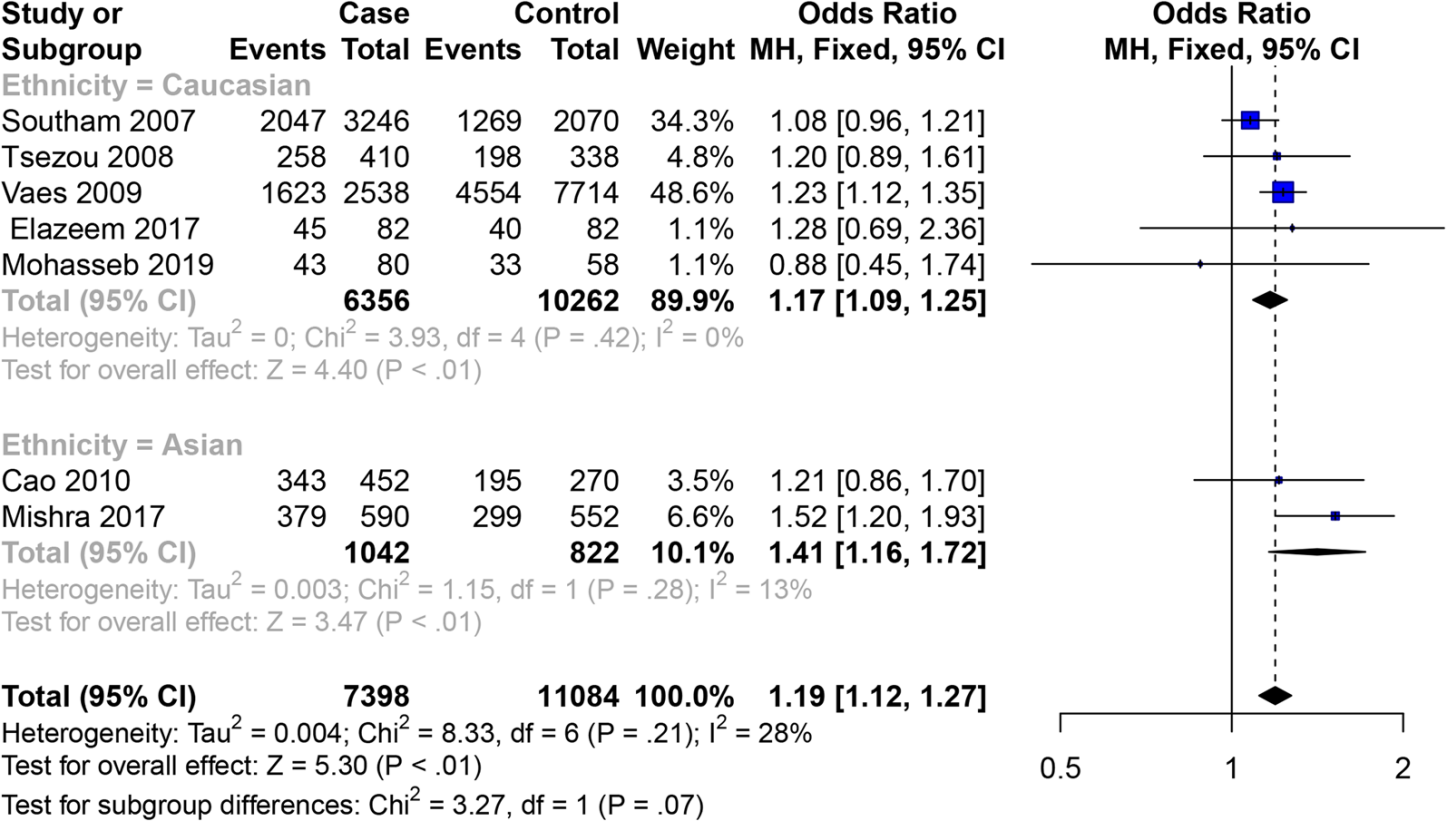
Forest plot of the correlation between GDF5 gene polymorphism and female OA of knee, hip and hand risk. Allele model (T vs. C)

**Fig. 9:**
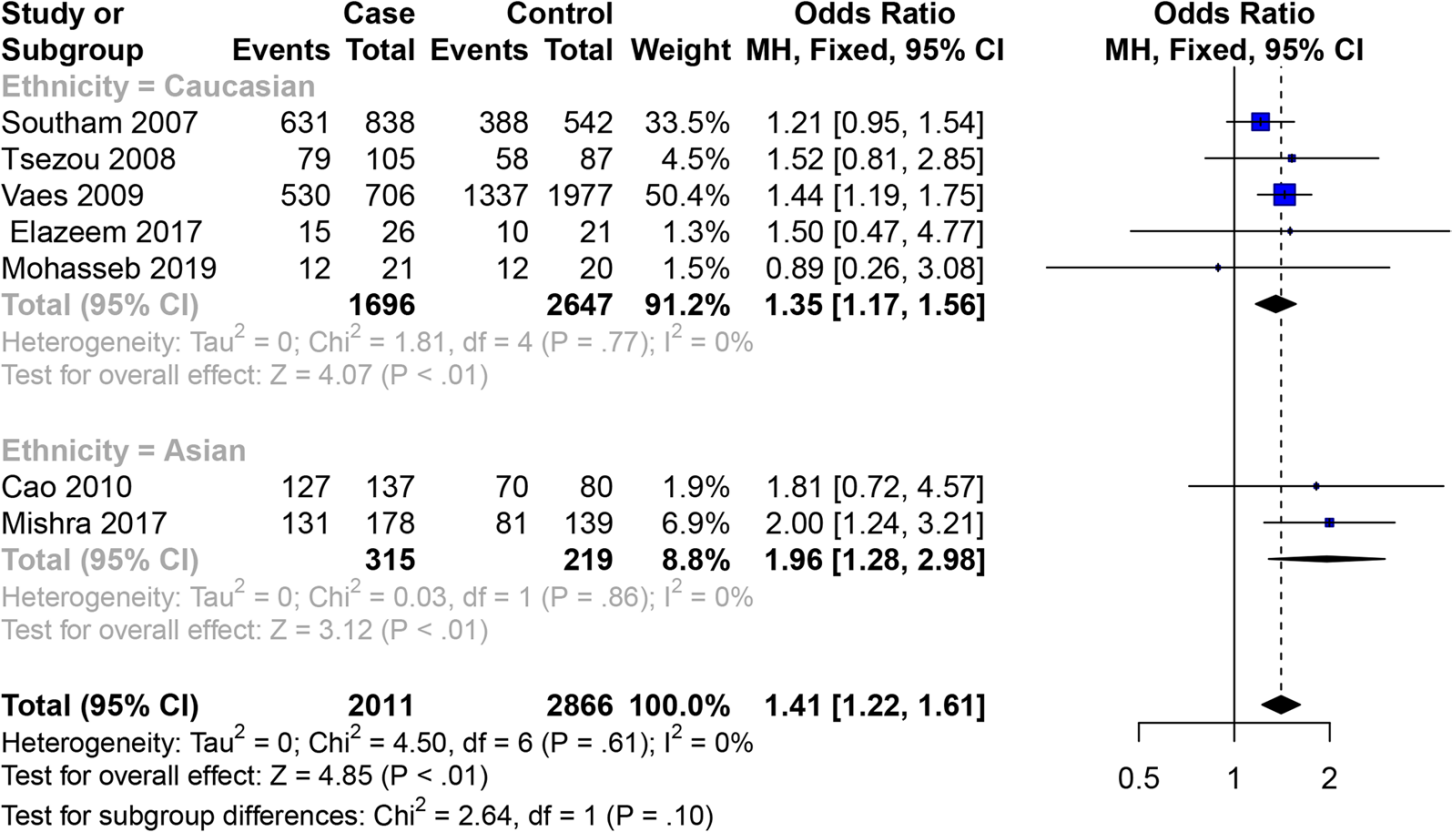
Forest plot of the correlation between GDF5 gene polymorphism and female OA of knee, hip and hand risk. Codominant model (TT vs. CC)

Eight articles studied the relativity between the GDF5 rs143383 SNP and female OA (Table 3), and the overall meta-analysis revealed that GDF5 rs143383 SNP allele model, codominant homozygote model and dominant model are related to the susceptibility to female OA, while there is no statistical significance in GDF5 rs143383 codominant heterozygote model. According to the results of subgroup analysis, allele model (OR = 1.41, 95% CI 1.16–1.72) and codominant homozygote model (OR = 1.96, 95% CI 1.28–2.98) are related to the susceptibility to OA in the Asian females, while it is no statistical significance in the codominant heterozygote model (OR = 1.14, 95% CI 0.76–1.72) and dominant model (OR = 1.46, 95% CI 0.99–2.14). The GDF5 rs143383 allele model OR = 1.17, 95% CI 1.09–1.25),codominant homozygote model (OR = 1.35, 95% CI 1.17–1.56), dominant model (OR = 1.23, 95% CI 1.07–1.40) and recessive model (OR = 1.22, 95% CI 1.11–1.35) are related to the susceptibility to OA in the Caucasian females, while there is no statistical significance in the codominant heterozygote model (OR = 1.14, 95% CI 0.99–1.31) (Figs. 8 and 9). The specific results are shown in Table 4.

### Heterogeneity and sensitivity analyses

Meta-analysis revealed heterogeneity, which may be explained by factors associated with HWE. However, HWE test (*P* > 0.05) was used to determine if subjects were selected reliably in each study, which showed a high level of reliability in each one. By using subgroup and the analysis of heterogeneity to determine the heterogeneity’s source, we found that Zhang et al. [26] were responsible for heterogeneity. We carefully analyzed this study and used the data provided in this paper, we calculated the OR and 95% CI, and however, we found that they were not consistent with the final results. We believe that data errors may be the main cause of heterogeneity, and the heterogeneity decreased after excluding the literature of Zhang et al. The sensitivity analysis of the included literature is carried out by using the method of excluding each study one by one, and the OR values of other studies are combined. It can be seen that the results are stable, indicating that the outcomes of Meta-analysis are believable. An analysis of subgroups was conducted, which no significant heterogeneity was detected in several studies of the Asian subgroup, indicating a good consistency. At the same time, we carried out a sensitivity analysis, and we finally found that the result is stable. We did not notice any significant changes in genotypes when we limited the number of high quality and HWE studies. The detailed results are shown in Table 4.

### Publication bias

**Fig. 10:**
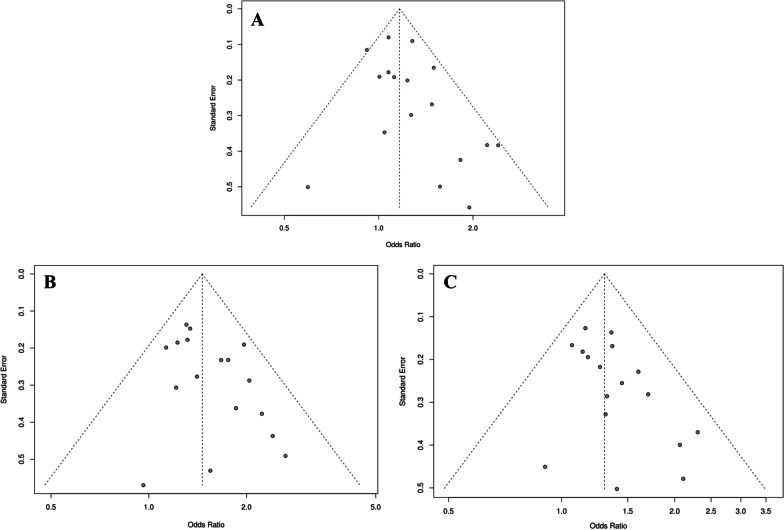
Funnel plot for publication bias among selected studies. **A** OA of knee, hip and hand codominant model (TC vs. CC). **B** Knee OA codominant model (TT vs. CC). **C** Knee OA dominant model (TT + TC vs. CC)

We performed funnel plots and Begg’s test to assess the publication bias of the literature. According to the funnel plots, publication bias was not evident **(**Fig. 10**)**. For statistical evidence, Begg’s tests were conducted and indicate that publication bias is not apparent.

## Discussion

The latest epidemiological study found that 8.1% of people over 45 years old had symptoms of OA, and the highest incidence rate was between 60 and 69 years old. And with the aging of the population becoming more and more serious, it is estimated that over 50-year-olds will account for 49% of the total population by 2050. If we do not conduct in-depth research in this direction, the proportion of elderly people suffering from OA will inevitably increase significantly [28]. Up to now, there is no effective drug to prevent or improve the progress of osteoarthritis in the whole world, and it is still focused on reducing pain and improving joint function [29, 30]. Severe OA seriously affects patients’ actions and pain, resulting in an extremely low happiness index. At present, operation is the main treatment method to eliminate pain and recover joint range of motion [31, 32]. At this stage, the cost of joint replacement surgery is relatively high. With the increasingly serious aging of the population, the social and economic burden of OA treatment will inevitably increase in the future. Therefore, the study on the pathogenesis of OA is urgent and has positive practical significance.

GDF5 participates in tissue differentiation and is related to the various bone-related diseases, which have been confirmed at the genetic level and through molecular biological mechanisms. It can provide help for early diagnosis of bone-related diseases and potential targets for treatment, and further understanding the mechanism of disease development, which can bring many new ideas to medical researchers [6, 33]. GDF5 regulates chondrogenesis through the canonical Wnt signaling pathway. Developmental dysplasia of the hip (DDH) is a multifactorial disease, which occurs under environmental and genetic influence. The genes displaying the most statistically significant co-expression link to GDF5. A specific polymorphism in GDF5 has been linked to DDH, and DDH patients more frequently carry the T allele [6, 34, 35]. This may be closely related to the occurrence and development of OA, possibly through the same mechanism. Studies in UK and Netherland have confirmed that the SNP of GDF5 rs143383 is closely correlated with the incidence rate of OA [11, 15, 16]. However, no correlation was found between OA and GDF5 gene polymorphisms in South Korea and Japanese populations [17, 18], which may be related to the genetic differences between eastern and western ancestors. This conclusion still lacks the correct guiding conclusion and has become the focus of the debate.

The previous meta-analysis data have not been updated [36], and some analyses have recorded identical data [37], which does not fully explain the relationship between GDF5 and OA. The Meta has carried out the most comprehensive analysis at present, analyzing different types of arthritis, gender and race. Although individual data are highly heterogeneous, which affects accurate conclusions, we have analyzed data with low heterogeneity and can still make effective summary because of the combination of numbers, so as to draw relatively more accurate conclusions.

The low heterogeneity gene model was selected for analysis throughout this meta. Based on the results, there was a significant correlation between GDF5 and OA in the total OA codominant heterozygote gene model in all populations with OA of the knee, hip, and hand, Asian populations and Caucasian populations, especially in Caucasian populations codominant homozygote gene model and dominant gene model.

As osteoarthritis differs in type, the correlation of knee osteoarthritis is the most obvious. The association between KOA and GDF5 can be evaluated based on an overall analysis of the four genotypes. In the overall allele gene, codominant homozygote gene, codominant heterozygote gene and dominant gene model, it is shown that there is a relationship between GDF5 gene and KOA. And it suggests the same results in both among Asians and Caucasians. In hip OA, it is generally explained that there is no significant relevance between GDF5 SNP and hip OA, and because of the high heterogeneity, this conclusion needs to be further verified, and the current study of hip OA is mainly Caucasian population and needs more data research and other ethnic groups. In hand OA, the overall analysis of the correlation between GDF5 and hand OA can be evaluated using these two genotypes. Codominant homozygote gene and dominant gene model suggest that GDF5 gene is associated with hand OA. However, the current study is focused on the Caucasian population, and the outcomes should be proved by an abundant case in other ethnic groups.

OA can arise naturally, with the aging population witnessing an increase in diagnoses of this pathology, but the root causes of OA have yet to be identified, and increasing interest is arising toward investigating biological sex as a risk factor. Clinical studies show increased prevalence and worse clinical outcomes for female patients [38, 39]. In the analysis of OA in different genders, we found that GDF5 was associated with OA in both males and females’ osteoarthritis. In males with OA, it is possible to reckon the association between GDF5 gene and OA using four genotypes. In the overall allele gene, codominant homozygote gene, codominant heterozygote gene and dominant gene model, the GDF5 gene is associated with OA, and the same results are suggested in the Caucasian population. In females with OA, three genotypes can be used to evaluate the relevance between GDF5 gene and OA. The overall allele gene, codominant homozygote gene and dominant gene model suggest that GDF5 gene is associated with OA, and the same results are suggested in Asian and Caucasian populations.

At present, it is generally believed that the pathogenesis of OA is due to the imbalance of cartilage tissue synthesis and catabolism, and the destruction of articular cartilage is difficult to repair, and the imbalance of chondrocytes synthesis and catabolism [40, 41]. Many factors are interrelated with the occurrence and progression of OA, such as joint composition and environmental factors, genetic predispositions, endocrine and metabolic diseases and mechanical injuries. Genetic factor is a significant pathogenic risk factor, and the genetic polymorphisms of multiple gene loci have been proved to be interrelated with the occurrence of OA in the region [42–45]. GDF5 belongs to the family of bone morphogenetic proteins (BMP), which is involved in bone growth and repair, such as the proliferation, differentiation, angiogenesis, and bone and cartilage formation. Research shows that GDF5 genetic polymorphisms are closely related to OA [46, 47]. However, it is not completely clear what role GDF5 plays in the occurrence and development of OA, and how it affects other signal pathways. The transmembrane serine/threonine kinases I and II can initiate GDF5’s signal cascade just like other BMPs. As a result of GDF5 binding, receptors are phosphorylated, the downstream Smad pathway is activated, and Smad is transferred to the nucleus to regulate gene transcription [48, 49]. Additionally, type I receptors bind both GDF5 and BMP2, and their complexes can recruit type II receptors, activating MAPK in the process [50]. Kan et al. [51] found that Sox11 (SRY-related HMG box11) transcription factor regulates the expression of GDF5, and Sox11 overexpressed in vitro and microsphere cell culture can directly activate the increase in GDF5 gene expression in chicken limb bud cells. The binding site of the Sox family is in the 5 ′—UTR region of GDF5 gene, indicating that Sox11 can specifically bind to this site, and the Sox11 can be used as a potential regulatory site of GDF5. However, the specific mechanism remains to be further studied and verified.

The main advantages of this study include: (1) Most of the studies contained in the meta-analysis are high quality case–control studies; (2) based on relatively large samples, we extract and analyze specific gene results. However, the current meta-analysis also has many limitations: (1) Although the meta-analysis contains a relatively large sample volume, it could still lead to overestimation and does not explain all the results; (2) the subjects of the study only include East Asian and Caucasian races and cannot reflect the overall situation. In subgroup analysis, the sample volume of every subgroup is smaller, which will also cause the analysis results to deviate from the actual situation; (3) publication bias and language bias caused by the unpublished results of some negative studies would affect the results of meta-analysis; (4) when patients’ informed consent is required in clinical research, and it comes to medical ethical issues, it is likely to result in low literature quality evaluations and inevitable biases, which will affect the reliability of meta-analysis conclusions; and (5) this paper only includes OA of knee joint, hip joint and hand joint, which is not all OA, and further data are needed to improve it. Therefore, the conclusion demands to be further confirmed by larger sample randomized controlled trials.

## Conclusion

Collectively, we concluded that the GDF5 rs143383 SNP has a significant relationship with the occurrence of OA in the whole population with OA of the knee, hip, and hand. From the analysis of each group, we got the same conclusion in KOA and hand OA, but which need further verification in hip OA. Considering gender, we found a close relationship between GDF5 rs143383 SNP and OA of the knee, hip and hand, both for men and women. This conclusion is more obvious in Caucasian people.

## Data Availability

In the article/supplementary material, you will find the original contributions; if you need further information, you may contact the authors.

## Abbreviations

CDMP-1: Cartilage-derived morphogenetic protein-1
BMP-14: Bone morphogenetic protein-14
SNP: Single nucleotide polymorphism
95% CI: 95% confidence interval
OR: Odds ratio
HWE: Hardy–Weinberg equilibrium
PRISMA: Preferred reporting items for systematic review and meta-analysis
